# Heterogeneous nuclear ribonucleoprotein (hnRNP) L promotes DNA damage-induced cell apoptosis by enhancing the translation of p53

**DOI:** 10.18632/oncotarget.17003

**Published:** 2017-04-10

**Authors:** Ji-Young Seo, Do-Yeon Kim, Seong-Hoon Kim, Hyo-Jin Kim, Hye Guk Ryu, Juhyun Lee, Kyung-Ha Lee, Kyong-Tai Kim

**Affiliations:** ^1^ Division of Integrative Biosciences and Biotechnology, Pohang University of Science and Technology (POSTECH), Pohang, Gyeongbuk, Republic of Korea; ^2^ Department of Life Sciences, Pohang University of Science and Technology (POSTECH), Pohang, Gyeongbuk, Republic of Korea; ^3^ Department of Pharmacology, School of Dentistry, Kyungpook National University (KNU), Daegu, Gyeongbuk, Republic of Korea; ^4^ Division of Bio-technology and Convergence, Daegu Haany University (DHU), Gyeongsan-si, Gyeongbuk, Republic of Korea

**Keywords:** p53, hnRNP L, apoptosis, IRES-mediated translation, post-transcriptional regulation

## Abstract

The tumor suppressor p53 is an essential gene in the induction of cell cycle arrest, DNA repair, and apoptosis. p53 protein is induced under cellular stress, blocking cell cycle progression and inducing DNA repair. Under DNA damage conditions, it has been reported that post-transcriptional regulation of p53 mRNA contributes to the increase in p53 protein level. Here we demonstrate that heterogeneous nuclear ribonucleoprotein (hnRNP) L enhances p53 mRNA translation. We found that hnRNP L is increased and binds to the 5’UTR of p53 mRNA in response to DNA damage. Increased hnRNP L caused enhancement of p53 mRNA translation. Conversely, p53 protein levels were decreased following hnRNP L knock-down, rendering them resistant to apoptosis and arrest in the G2/M phase after DNA damage. Thus, our findings suggest that hnRNP L functions as a positive regulator of p53 translation and promotes cell cycle arrest and apoptosis.

## INTRODUCTION

The p53 gene is the most powerful of the tumor suppressor genes [1]. It plays a critical role in DNA repair, cell cycle arrest, apoptosis, and senescence, by inducing transcription of downstream genes, such as p21, Puma, and Bax [2–5]. It was reported that mutations of the p53 gene occur in about 50% of human cancers [6]. This demonstrates the importance of p53 inhibition in tumorigenesis, and how significant the normal function of p53 is for anti-cancer effects.

Under normal conditions, p53 protein has a short half-life, and is maintained at low levels in the cell. However, p53 protein is induced and activated for appropriate function following numerous stresses, including DNA damage, oxidative stress, and nutrient depletion [7–9]. Many studies have demonstrated that the stabilization and activation of p53 protein are mainly controlled by post-translational regulatory mechanisms [10, 11]. However, post-transcriptional regulation is also important for finely tuning p53 expression levels in both normal and DNA damage conditions. Especially, it has been reported that the mechanism of protein synthesis is a critical regulation point for the induction of p53 protein under cell stress conditions, including DNA damage. While irradiation or DNA-damaging reagents cause the accumulation of p53 protein, addition of the translation inhibitor cycloheximide blocks the induction of p53 protein, implicating the importance of translation on p53 protein accumulation [12, 13]. Translation of p53 mRNA is regulated by some miRNAs and RNA-binding proteins. p53 translation negative regulators including miR-125b, miR-25 and nucleolin inhibit p53 expression and positive regulators including HuR and heterogeneous nuclear ribonucleoprotein (hnRNP) Q enhance p53 expression and p53-mediated apoptosis and cell cycle arrest [12, 14–18].

In the translation process, translation initiation is the rate-limiting step [19, 20]. Translation initiation occurs by cap-dependent and cap-independent mechanisms [21]. Cap-independent translation occurs by a mechanism of direct recruitment of ribosome subunits, called internal ribosome entry site (IRES)-mediated translation. IRES-mediated translation is associated with diverse cellular conditions like cell differentiation, proliferation, circadian rhythm, and apoptosis [22–24]. In particular, it has been reported that several proteins involved in cellular stress are synthesized by IRES-mediated translation because cap-dependent translation is blocked under cellular stress conditions [25]. In numerous stress conditions, p53 gene expression must be enhanced to activate the pathway for DNA repair and cell death. Thus, under stress conditions, p53 mRNA can be translated in an IRES-mediated manner [26, 27]. The 5’UTR of p53 mRNA has an IRES element, and its IRES activity is enhanced by several IRES *trans*-acting factors (ITAFs) [28, 29]. Inhibition of ITAFs decreases the translation rate of p53 mRNA and p53-mediated apoptosis [17], supporting IRES-mediated translation as an important mechanism in p53 regulation.

Heterogeneous nuclear ribonucleoprotein (hnRNP) L is an RNA-binding protein enriched in the nucleus. hnRNP L binds to CA-repeat and CA-rich RNA elements and plays an important role in post-transcriptional regulatory mechanisms like pre-RNA splicing, mRNA degradation, mRNA export, and IRES-mediated translation [30–34]. It can translocate between the nucleus and cytosol in response to stress stimulation such as hypoxia, and the localization of hnRNP L is critical for its specific function [35]. According to catalogue of somatic mutation in cancer (COSMIC) database, hnRNP L gene is mutated in cancers. It suggests that mutated hnRNP L may be exploited by cancer to lead to tumorigenesis. Here, we provide evidence that hnRNP L is a new ITAF for the translation of p53 mRNA. It associates with the 5’UTR of p53 mRNA, and functions as a positive regulator of p53 translation. Moreover, we show that hnRNP L knock-down blocks protein synthesis of p53, which inhibits cell apoptosis and cell cycle arrest induced by treatment with the DNA-damaging drug etoposide.

## RESULTS

### DNA damage-induced hnRNP L enhances the induction of p53 protein

**Figure 1:**
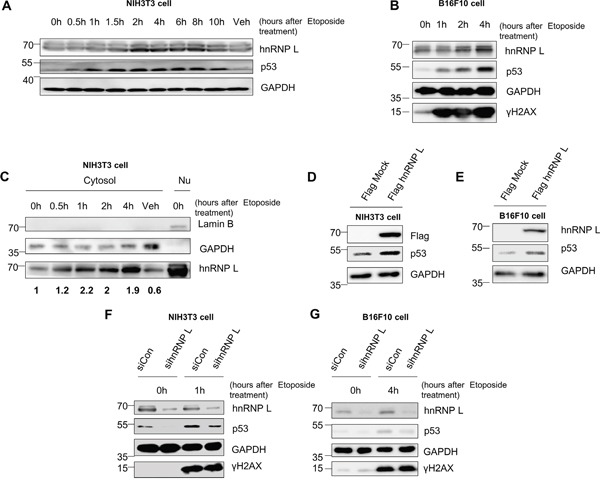
Accumulation of p53 is enhanced by an increase in hnRNP L after DNA damage-inducing drug treatment **(A, B)** Endogenous mouse p53 and hnRNP L are induced after etoposide treatment. **(A)** NIH3T3 and **(B)** B16F10 cells were treated with 100 μM etoposide for the indicated times. Treatment with dimethylsulfoxide (DMSO) for 10 h was used as vehicle control (Veh). The levels of endogenous proteins were analyzed by western blotting (WB) using anti-p53, anti-GAPDH, anti-hnRNP L and anti-γH2AX antibodies. The housekeeping protein, GAPDH was used as loading control. **(C)** Protein level of cytosolic hnRNP L increases after treatment with etoposide. NIH3T3 cells were fractionated into cytosol and nucleus after exposure to 100 μM etoposide. hnRNP L protein levels of fractionated cytosolic lysate were determined by WB using anti-hnRNP L antibody. GAPDH protein was used as loading control and cytosol marker. Lamin B protein was analyzed as nucleus marker. DMSO treatment for 4 h was used as vehicle control. Nu, Nuclear lysate. The numbers at the bottom mean the fold increases relative to control. The amount of hnRNP L was normalized to GAPDH. **(D, E)** hnRNP L overexpression results in increased p53 protein. Flag-tagged hnRNP L was transfected on **(D)** NIH3T3 and **(E)** B16F10 cells. Flag-tagged hnRNP L overexpression was confirmed by WB using anti-Flag antibody. **(F, G)** Induction of p53 is impaired on both **(F)** NIH3T3 and **(G)** B16F10 cells by knock-down of hnRNP L after 100 μM etoposide treatment. Knock-down of hnRNP L was confirmed by WB using anti-hnRNP L antibody.

It is well-known that the accumulation of p53 protein after treatment with the DNA-damaging drug etoposide is induced through increases in both protein stability and protein synthesis [17, 36]. Accumulation of p53 protein in nontumorigenic mouse fibroblast NIH3T3 and mouse melanoma B16F10 cells was detectable after treatment with etoposide (Figure 1A, 1B). However, the amount of p53 mRNA remained stable in the total cell extracts and cytosolic lysates (Supplementary Figure 1A, 1B and 1C). To investigate the possible factors that might regulate the accumulation of p53 protein under cell stress conditions, we used the RNA-binding protein (RBP)-target interaction prediction web server, RBPmap (http://rbpmap.technion.ac.il/). Among the several candidate factors which are expected to bind to p53 mRNA, hnRNP L was reported to be involved in regulation of genes related to cell stress and cancer [34, 35]. Moreover, we found that hnRNP L expression is changed in the presence of etoposide. The level of hnRNP L protein increased 2 hours after etoposide treatment (Figure 1A, 1B). In NIH3T3 cells treated with etoposide, hnRNP L expression in the cytoplasmic fraction increased within 30 minutes (Figure 1C), suggesting that hnRNP L may control p53 expression. To confirm this possibility, we investigated the effect of hnRNP L on the expression and induction of p53. Interestingly, overexpression of flag-tagged hnRNP L upregulated the level of p53 protein in both NIH3T3 and B16F10 (Figure 1D, 1E). Moreover, knock-down of hnRNP L suppressed accumulation of p53 protein, both in normal and DNA-damaged cells (Figure 1F, 1G). These results show that DNA damage-induced hnRNP L positively regulates the expression of p53.

### hnRNP L increases translation of p53 mRNA

**Figure 2:**
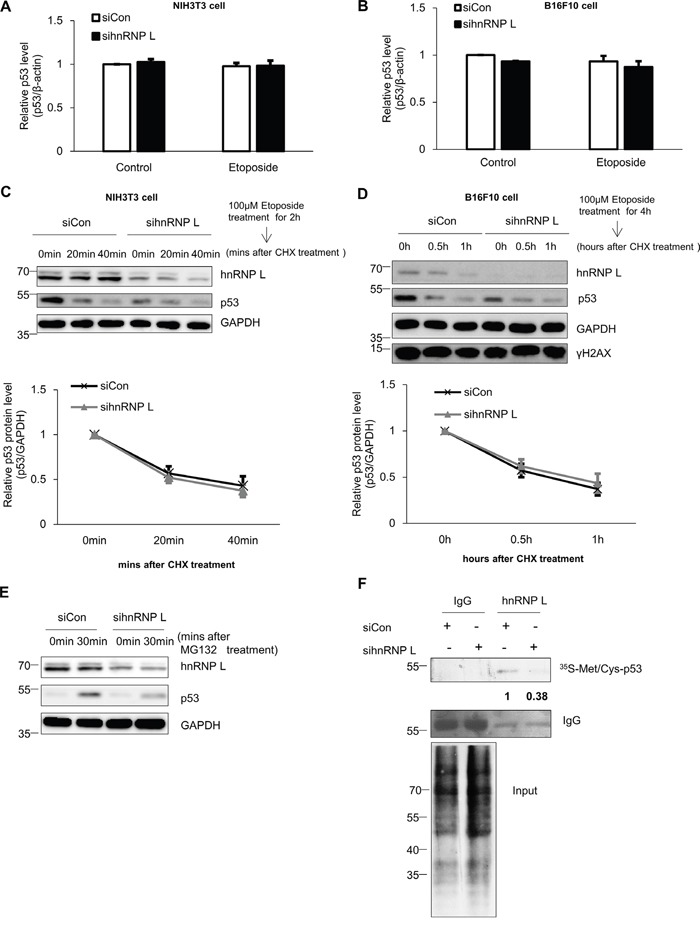
hnRNP L controls the expression of p53 through translational regulation **(A, B)** hnRNP L does not affect endogenous p53 mRNA levels in either normal or DNA-damaged cells. Control siRNA or hnRNP L siRNA was transfected into **(A)** NIH3T3 and **(B)** B16F10 and cells treated with 100 μM etoposide for 1 hour and 4 hours, respectively. Endogenous p53 mRNA levels were analyzed by quantitative real-time PCR (qRT-PCR) and normalized to β-actin. The bars represent the mean±SEM (n=3). **(C, D)** Knock-down of hnRNP L does not affect p53 protein stability. After 100 μM etoposide was added to **(C)** NIH3T3 and **(D)** B16F10 cells, 50 μg/ml cycloheximide (CHX) was then added for the indicated times. Endogenous p53 levels and knock-down of hnRNP L were determined by WB. The amount of p53 protein was normalized to GAPDH. p53 protein levels of 0 time point and control siRNA transfected cells were set as 1. Data show relative p53 protein intensity from four independent experiments (mean±SEM). **(E)** hnRNP L increases the translation rate of p53 mRNA. After transfection with control or hnRNP L siRNA, 10 μM MG132 was added for the indicated times. Changes in the levels of p53 protein by translation or knock-down were assessed by WB. **(F)** Metabolic labeling shows that reduction of hnRNP L downregulates protein synthesis of p53. After transfection of NIH3T3 cells with control or hnRNP L siRNA, cells were incubated in medium containing ^35^S-labeled methionine (^35^S-Met) and ^35^S-labeled cysteine (^35^S-Cys) and 10 μM MG132. Newly synthesized p53 proteins were detected after immunoprecipitation (IP) with monoclonal p53 antibody. The numbers at the bottom of the first lane mean the fold increases relative to control. Data information: In **(A-D)**; Two-way ANOVA

To define the precise role of hnRNP L on p53 regulation, we tested whether hnRNP L contributes to p53 protein accumulation through enhanced transcription or mRNA stabilization. First, we investigated the effect of hnRNP L silencing on endogenous p53 mRNA levels. The reduction of hnRNP L did not change the levels of p53 mRNA in NIH3T3 and B16F10 cells treated with or without etoposide (Figure 2A, 2B). This result means that the decline in p53 protein accumulation in the hnRNP L-silenced cell is not due to reduced levels of p53 mRNA. Many studies have reported that the stability of p53 protein is increased in various cellular stress conditions, and is important for its accumulation [11, 37]. Therefore, we investigated p53 protein stability after knock-down of hnRNP L. We measured p53 protein stability in control and hnRNP L siRNA transfected etoposide-treated NIH3T3 cells with cycloheximide treatment. The levels of p53 protein were reduced rapidly and to a similar degree in both cells after treatment with cycloheximide (Figure 2C, 2D). These results indicate that hnRNP L has little or no effect on p53 protein stability in both NIH3T3 and B16F10 cells.

Based on previous reports of the importance of translation for p53 protein accumulation, we also investigated the contribution of the translation process on the accumulation of p53 in the presence of the DNA-damaging drug, etoposide [12, 17]. Addition of the transcription inhibitor actinomycin D did not affect the accumulation of p53 protein, whereas treatment with cycloheximide following etoposide treatment completely diminished the induction of p53 protein (Supplementary Figure 2A). Therefore, we tested whether hnRNP L increases the translation efficiency of p53 mRNA. The rate of p53 protein synthesis was examined in control and hnRNP L siRNA transfected cells after treatment with MG132, a proteasome inhibitor. Knock-down of hnRNP L led to a dramatic decline in p53 protein accumulation, suggesting the possibility that hnRNP L facilitates the translation of p53 mRNA (Figure 2E). Next, we measured newly synthesized p53 proteins in metabolically labeled NIH3T3 cells transfected with control or hnRNP L siRNA. Compared to control transfected cells, hnRNP L knock-down decreased *de novo* p53 protein synthesis, without changing total protein levels in the cell. This strongly suggests that hnRNP L functions to promote the translation rate of p53 mRNA (Figure 2F).

### IRES activity of p53 mRNA is enhanced by hnRNP L

**Figure 3:**
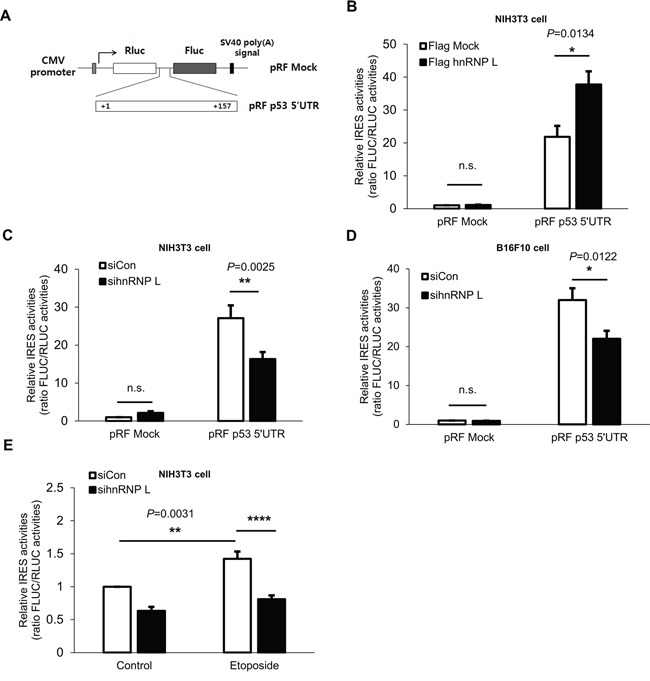
hnRNP L enhances IRES activity of p53 5’UTR **(A)** Schematic representation of the bicistronic luciferase pRF plasmids used for the detection of p53 5’UTR IRES activity. The 157bp p53 5’UTR was inserted between the two cistrons, *Renilla* luciferase (RLUC) and firefly luciferase (FLUC). **(B)** IRES activity of p53 5’UTR is increased under hnRNP L overexpression. NIH3T3 cells were transfected with flag Mock or flag hnRNP L and 24 h later with pRF mock vector or pRF p53 5’UTR vector. Luciferase activity is shown as the ratio of FLUC to RLUC and IRES activity of pRF mock and flag Mock transfected cells was set as 1. The bars represent the mean±SEM (n=3). **(C, D)** Suppression of p53 5’UTR IRES activity is observed after knock-down of hnRNP L. At 24 h after transfection with control or hnRNP L siRNA, pRF mock or pRF mp53 5’UTR vector was transfected into **(C)** NIH3T3 and **(D)** B16F10 cells. IRES activity of pRF mock and control siRNA transfected cells was set as 1. The bars represent the mean±SEM (n=7, n=4). **(E)** Increase of p53 5’UTR IRES activity under etoposide treatment is diminished through the reduction of hnRNP L. Cells were transfected with pRF p53 5’UTR at 24 h after transfection with either control or hnRNP L siRNA, and DMSO or etoposide was added at 18 h after transfection. IRES activity of control siRNA transfected and DMSO treated NIH3T3 cells was set as 1. The bars represent the mean±SEM (n=7). Data information: In **(B–E)**, n.s., non-significant, *P<0.05, **P<0.01, ****P<0.0001(Two-way ANOVA)

It has been shown that IRES-mediated translation makes an important contribution to p53 protein synthesis [17, 38]. In addition, several studies have reported that under conditions of stress, IRES-mediated translation of p53 mRNA is enhanced, and contributes to the elevation of p53 protein levels [13]. The importance of IRES-mediated translation of p53 mRNA was confirmed by our next experiment. When the cells were treated with rapamycin and etoposide, only a slight decline of p53 induction was observed, unlike the case with cycloheximide (Supplementary Figure 2B). Rapamycin was used to inhibit cap-dependent translation as a main inhibitor of mTOR and the activity was confirmed by measuring phosphorylation status of S6 ribosome proteins. This result means that cap-independent translation of p53 mRNA is needed for p53 protein induction. Moreover, since it was reported that hnRNP L functions as an ITAF enhancing the IRES-mediated translation of Cat-1 [34], we investigated further whether hnRNP L enhances the IRES-mediated translation of p53 by utilizing a pRF bicistronic luciferase vector. Because the IRES-mediated translation of p53 mRNA is induced through the 5’UTR, the 5’UTR of mouse p53 was inserted into the vector between the *Renilla* luciferase (RLUC) and firefly luciferase (FLUC) cistrons (Figure 3A). RLUC translation is cap-dependent, whereas FLUC translation is cap-independent. The IRES activity is calculated by the ratio of FLUC to RLUC. As reported earlier, IRES activities are dramatically increased by mouse p53 5’UTR [17]. When hnRNP L was overexpressed, the IRES activities were enhanced by about 70% (Figure 3B). Conversely, when the hnRNP L level was reduced, IRES activities of p53 5’UTR declined significantly in both NIH3T3 and B16F10 cells (Figure 3C, 3D). This observation validated the functional role of hnRNP L as an ITAF that enhances the translation of p53 mRNA. Next, we evaluated the impact of hnRNP L on IRES-mediated translation of p53 mRNA under stress conditions. We transfected the pRF p53 5’UTR vector into control siRNA and hnRNP L siRNA transfected cells, and subsequently treated them with control solvent (DMSO) or etoposide. As expected, in the control siRNA transfected cells, IRES-mediated translation of p53 mRNA increased by about 40% in cells incubated with etoposide compared to cells treated with DMSO. However, such a rise in IRES activities was diminished by knock-down of hnRNP L (Figure 3E). These data indicate that hnRNP L plays a key factor to increase IRES-mediated translation of p53 mRNA and accumulation of p53 protein under normal and DNA-damaging conditions. We previously found that hnRNP Q functions as an ITAF of p53 mRNA [17]. To test whether hnRNP L and hnRNP Q affect each other's function for the IRES-mediated translation of p53 mRNA, we analyzed the IRES activities of p53 5’UTR under knock-down of hnRNP L and hnRNP Q. p53 IRES activity in cells transfected with both sihnRNP Q and sihnRNP L was comparable with that in cells transfected with either sihnRNP Q or sihnRNP L (Supplementary Figure 3A). This result reveals that hnRNP L and hnRNP Q work closely together to enhance the IRES-mediated translation of p53 mRNA.

### hnRNP L functions as an ITAF by binding to p53 mRNA

**Figure 4:**
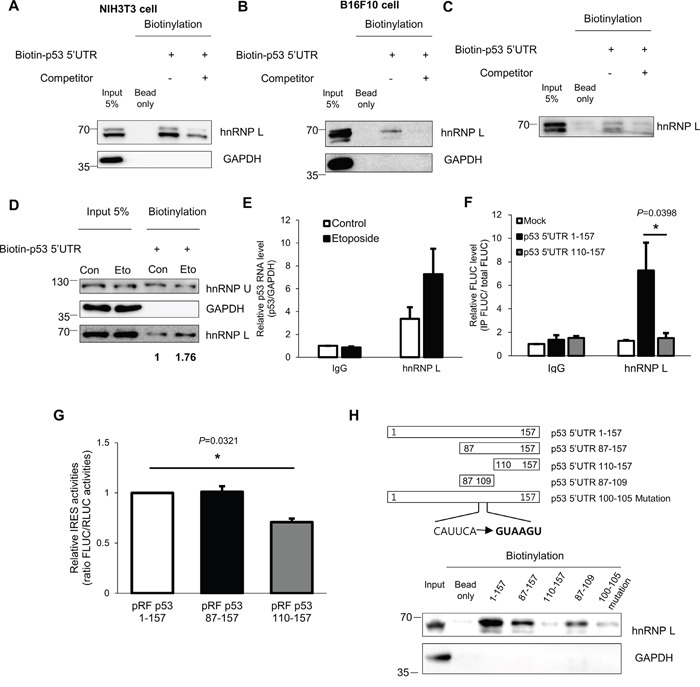
hnRNP L interacts with p53 mRNA and the binding apparently increases after DNA damage **(A, B)** *In vitro* binding assays were performed by incubating *in vitro* transcribed biotin-p53 5’UTR with **(A)** NIH3T3 or **(B)** B16F10 cell extracts and followed by pull down with streptavidin beads. The binding between p53 5’UTR and hnRNP L was confirmed by Western blotting. GAPDH was used as negative control. Non-biotinylated p53 5’UTR was used as competitor. **(C)** hnRNP L directly binds to p53 5’UTR. Purified hnRNP L proteins were incubated with *in vitro* transcribed biotin-p53 5’UTR. **(D)** Under DNA damage conditions, the amount of hnRNP L proteins interacting with p53 5’UTR increases. *In vitro* transcribed biotin-p53 5’UTR was incubated with cytoplasmic extracts of non-treated (Con) or etoposide-treated (Eto) NIH3T3 cells. hnRNP U was used as negative control and GAPDH was used as loading and negative control. The numbers at the bottom mean the fold increases relative to control. **(E)** Endogenous hnRNP L binds endogenous p53 mRNA and the binding increases under etoposide treatment. Lysates of non-treated (Control) and etoposide treated (Etoposide) NIH3T3 cells were used for RNA-immunoprecipitation (RNAIP) analysis using IgG control and hnRNP L antibody. RNA abundance in IP samples was determined by qRT-PCR. The levels of p53 mRNA were normalized to GAPDH mRNA levels. p53 mRNA level in control-IgG sample was set as 1. The bars represent the mean±SEM (n=3). **(F)** hnRNP L binds to p53 5’UTR 1-109 region. pRF Mock, pRF p53 5’UTR 1-157 or pRF p53 5’UTR 110-157 vector was transfected into NIH3T3 cells. 24h later, cells were lysed and the lysates were used for RNAIP using control IgG and hnRNP L antibody. RNA abundance was determined by qRT-PCR. The levels of FLUC mRNA in IP samples were normalized to input FLUC mRNA levels. FLUC mRNA level in control IgG sample of the pRF Mock transfected cells was set as 1. The bars represent the mean±SEM (n=4). **(G)** The region between nucleotides 87 and 109 of p53 5’UTR is important for IRES activity of p53 5’UTR. To confirm IRES activities of serial deletion constructs, luciferase assay was carried out. Luciferase activity is shown as the ratio of FLUC to RLUC. IRES activity of p53 5’UTR 1–157 full length construct was set as 1. The bars represent the mean±SEM (n=3). **(H)** To identify the binding region of hnRNP L to the 5’UTR of p53 mRNA, *in vitro* binding assays were conducted. Biotin-labeled p53 5’UTR constructs were incubated with NIH3T3 cell extracts. The interaction of p53 5’UTR and hnRNP L was verified by Western blotting. GAPDH was used as negative control. Data information: In (E-G), *P<0.05 (Two-way ANOVA).

The binding of hnRNP L to the 5’UTR of p53 was investigated using an *in vitro* binding assay. We found that hnRNP L was bound by biotinylated-p53 5’UTR, and this binding was reduced by competition with unlabeled-p53 5’UTR (Figure 4A, 4B). In the control, the binding of GAPDH to the p53 5’UTR was undetectable. Thus, the interaction of hnRNP L with the 5’UTR of p53 mRNA is a specific interaction. Moreover, to verify that the interaction occurs in the cytoplasm where translation of mRNAs occurs, biotinylated-p53 5’UTR transcripts were incubated with either cytoplasm or nucleus fraction from NIH3T3 cells. We observed that the p53 5’UTR associates with hnRNP L in both cytoplasm and nucleus (Supplementary Figure 4A). When we tested whether coding region and 3’UTR of p53 mRNA bind to hnRNP L, hnRNP L preferentially binds to 5’UTR and 3’UTR rather than coding region of p53 mRNA (Supplementary Figure 4B). Next, to confirm whether binding of hnRNP L and 5’UTR of p53 mRNA is direct or indirect, we performed an *in vitro* binding assay using purified hnRNP L protein and biotinylated-p53 5’UTR. We verified that the association of hnRNP L and p53 5’UTR is direct (Figure 4C). Furthermore, to confirm the effect of DNA damage on the binding of hnRNP L to the p53 5’UTR, we performed *in vitro* binding assays using cytoplasmic extracts of NIH3T3 cells treated with etoposide. As a result, the association of hnRNP L and p53 5’UTR became stronger under conditions of DNA damage (Figure 4D). Next, we confirmed an interaction between endogenous hnRNP L and p53 mRNA using RNA-immunoprecipitation (RNAIP). hnRNP L antibody enabled us to have immunoprecipitation of p53 mRNA about 3.5-fold compared with control IP reactions using IgG. Under stressed condition, the association of hnRNP L and p53 mRNA was increased (Figure 4E). We tried to identify the region within the p53 mRNA 5’UTR that contributes to hnRNP L binding using the pRF vectors containing serially deleted 5’UTR of p53 (Figure 4F). The chimeric reporter vectors were transfected into NIH3T3 cells and RNAIP was performed using hnRNP L antibody and control IgG. This analysis showed that hnRNP L preferentially binds to the p53 5’UTR 1-157 chimeric transcripts, while the interaction with p53 5’UTR 110-157 chimeric transcripts is weak. This result suggests that the region between nucleotides 87 and 109 within p53 5’UTR is important for association of hnRNP L. The results of the binding assay correlated with the decrease in the IRES activity test using the pRF bicistronic vector containing serially deleted nucleotides (Figure 4G). To confirm the interaction reciprocally, hnRNP L was pulled down by serially deleted biotinylated-p53 5’UTR transcripts. As a consequence, the binding of hnRNP L with p53 5’UTR disappeared in the 109 nucleotides deleted construct, supporting that the binding region of hnRNP L lies between nucleotides 87 and 109 of the p53 5’UTR (Figure 4H). hnRNP L was reported to preferentially bind to CA-rich regions, and to be involved in the regulation of RNA containing CA-rich elements [39]. Interestingly, examination of the p53 5’UTR sequence revealed a region between nucleotides 100 and 105 with the sequence CAUUCA which is reported to interact strongly with hnRNP L (Supplementary Figure 4C) [40]. This sequence was considered as the binding site for hnRNP L. To test this hypothesis, we generated p53 5’UTR mutant containing GUAAGU substitution instead of CAUUCA sequences. We found that CAUUCA sequences is an important binding region of hnRNP L as p53 5’UTR mutant binds very weakly to hnRNP L (Figure 4H). Taken together, hnRNP L binds to p53 5’UTR and positively regulates p53 translation.

### Reduction of hnRNP L suppresses cell cycle arrest and cell apoptosis induced by DNA damage

**Figure 5:**
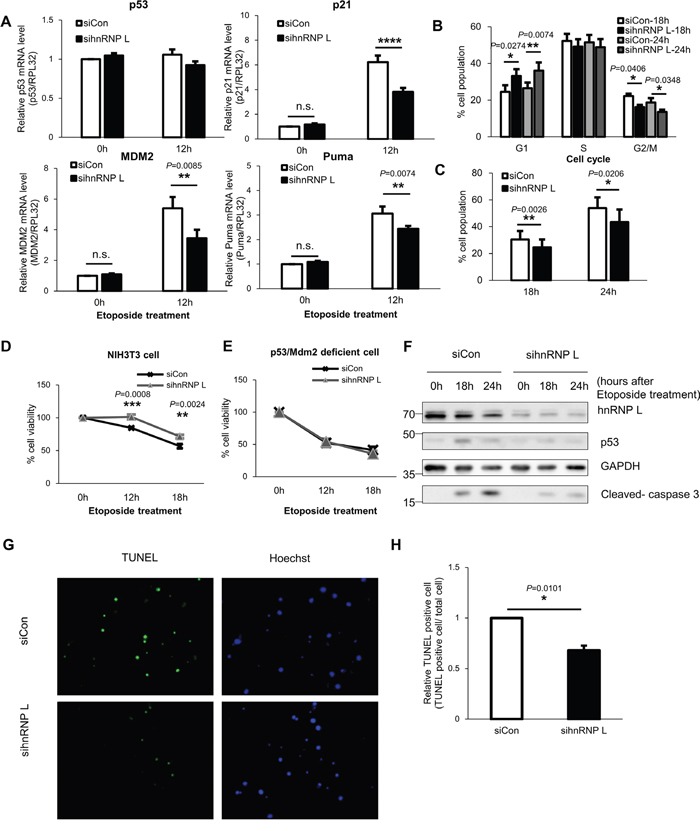
Reduction of hnRNP L downregulates p53 expression and relieves cell cycle arrest and DNA damage-induced apoptosis of NIH3T3 cells **(A)** mRNA levels of p53 target genes including p21, Mdm2 and Puma decrease in NIH3T3 cells transfected with hnRNP L siRNA and under etoposide treatment. At 24 h after transfection with control or hnRNP L siRNA, NIH3T3 cells were treated with or without 100 μM etoposide for 12 h. The levels of p21, MdmM2 and Puma mRNAs were analyzed by qRT-PCR and normalized to RPL32 mRNA levels. mRNA levels in control siRNA transfected and non-etoposide treated cells were set as 1. The bars represent the mean±SEM (n=5). **(B, C)** hnRNP L silencing lowers p53-mediated G2/M arrest and cell death. At 24 h after transfection with control or hnRNP L siRNA, NIH3T3 cells were treated with 50 μM etoposide for the indicated times and stained with DNA dye, propidium iodide (PI). The data were analyzed by flow cytometry. The bars represent the mean±SEM (n=4). **(D)** Reduction of hnRNP L increases cell viability. Control siRNA or hnRNP L siRNA transfected cells were exposed to 50 μM etoposide for the indicated times and the cell viability was assessed by MTT assay. The graph represents the mean±SEM (n=3). **(E)** In immortalized fibroblasts from p53/Mdm2 double-knockout mouse, hnRNP L does not affect cell viability. 50 μM etoposide was added to p53/Mdm2 double-knockout mouse fibroblasts transfected with siCon or sihnRNP L and MTT assay was conducted for measurement of cell viability. The graph represents the mean±SEM (n=3). **(F)** p53 expression is suppressed by knock-down of hnRNP L, which reduces activation and cleavage of caspase 3. Transfected cells were treated with 50 μM etoposide for the indicated times. Knock-down of hnRNP L was confirmed by WB. **(G, H)** Cell apoptosis was suppressed by hnRNP L knock-down. TUNEL assay was performed in cells transfected with control or hnRNP L siRNA and treated with 100 μM etoposide for 48 h. Nuclei were stained with Hoechst 33342. **(G)** Representative image from four independent experiments. **(H)** The diagram shows relative apoptotic cells measured by TUNEL assay. The bars represent the mean±SEM (n=4). More than 700 cells were analyzed in both group. The number of TUNEL-positive cells in control siRNA transfected cells was set as 1. Data information: In **(A-E, H)**, *P<0.05, **P<0.01, ***P<0.001, ****P<0.0001 (Two-way ANOVA, Student's t-test).

p53 gene is well-known to regulate cell cycle progression and apoptosis by functioning as a transcription factor [3]. In this study, we have confirmed the reduction of p53 protein by hnRNP L silencing. Therefore, we determined whether the mRNA levels of p53 downstream target genes are also decreased by knock-down of hnRNP L. NIH3T3 cells were transfected with control or hnRNP L siRNA, and then incubated with or without etoposide. When NIH3T3 cells were exposed to etoposide, the mRNA levels of p53 target genes, p21, Mdm2, Puma, increased compared to the untreated cells (Figure 5A). Under normal conditions, hnRNP L knock-down had no effect on expression of each target gene. However, in cells exposed to the DNA-damaging agent, knock-down of hnRNP L resulted in reduced mRNA expression of p21, Mdm2 and Puma compared to control siRNA transfected cells. This result suggests the possibility that hnRNP L knock-down may control cell cycle and DNA-damaging drug-induced apoptosis because p21 is a key regulator controlling cell cycle, and Puma is a proapoptotic gene [41, 42]. Therefore, we tested whether p53 accumulation lowered by hnRNP L knock-down affects cell cycle arrest in cell stress conditions. In the presence of etoposide, the cell cycle was arrested at S phase and G2/M as reported previously (Figure 5B and Supplementary Figure 5A) [18]. Cell cycle analysis showed that there were fewer cells at G2/M phase in hnRNP L siRNA transfected cells than in control cells, though no difference was seen at S phase. Moreover, though extended treatment with etoposide led to accumulation of more cells at sub G1, an indicator of apoptosis-induced DNA fragmentation, the number of cells at sub G1 was reduced when hnRNP L was knocked down, indicating that decreased hnRNP L expression alleviates cell death (Figure 5C and Supplementary Figure 5A). Therefore, reduction of hnRNP L seems to relieve cell cycle arrest in G2/M phase and decrease cell death. The effect of hnRNP L on cell death was also determined by MTT assay. At 12 hours and 18 hours after exposd to etoposide, cell viability increased by about 15% by silencing of hnRNP L (Figure 5D). To confirm that the increased cell viability by reduced hnRNP L was a p53-mediated outcome, MTT assays were carried out on immortalized fibroblasts from a p53/Mdm2 deficient mouse. hnRNP L knock-down had no effect on cell survival in the absence of p53, indicating that hnRNP L reduces cell viability by increasing the level of p53 protein (Figure 5E). Next, to determine that the effect of hnRNP L on DNA damage-induced apoptosis was not due to necrosis, activation of caspase3, an executioner caspase, was analyzed and terminal deoxynucleotidyl transferase dUTP nick end labeling (TUNEL) assays were performed. Our results show that the active (cleaved) form of caspase3 was decreased in cells with decreased hnRNP L (Figure 5F). Moreover, we confirmed that lowered hnRNP L expression attenuated etoposide induced apoptosis, which was verified by the reduced number of TUNEL-positive cells (Figure 5G, 5H). Taken together, these data indicate that hnRNP L elevates DNA damage-induced apoptosis.

## DISCUSSION

**Figure 6:**
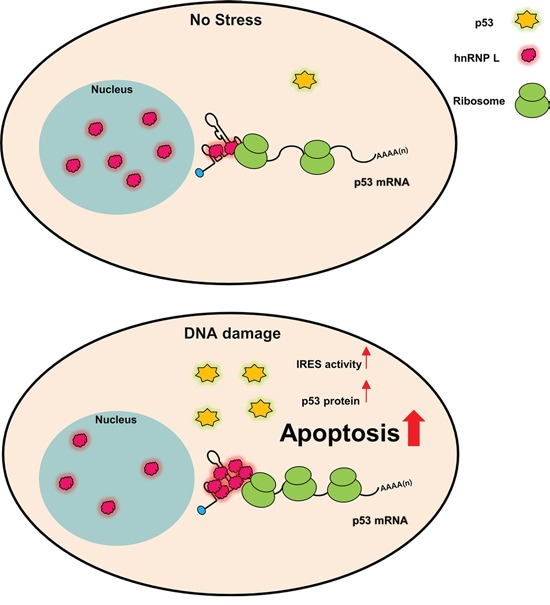
Model for p53 IRES-mediated translation activated by hnRNP L in normal and DNA damage conditions hnRNP L positively regulates IRES-mediated translation of p53 mRNA. Under DNA damage condition, hnRNP L translocates from the nucleus to the cytoplasm in early times and hnRNP L expression also increases. Increased hnRNP L elevates IRES-mediated translation of p53 mRNA and increased p53 proteins induce apoptosis in DNA damaged cells.

In this study, we have identified that hnRNP L increases the IRES-mediated translation of p53 mRNA. Etoposide treatment promoted hnRNP L accumulation and this contributed to the induction of p53. hnRNP L activates the translation of p53 mRNA by binding each other and this binding is increased in response to DNA damage. We found that the reduction of hnRNP L expression blocks the translation of p53 mRNA and the accumulation of p53 protein. Along with this, decreased hnRNP L alleviated DNA damage-mediated apoptosis and cell cycle arrest at G2/M phase (Figure 6).

Etoposide treatment triggered an increase in hnRNP L expression that could be observed in the cytoplasm 30 minutes after treatment (Figure 1A, 1B and 1C). This suggests the possibility that hnRNP L translocates from the nucleus to the cytoplasm in response to DNA damage. In fact, it has been suggested that hnRNP L carries a nuclear localization signal (NLS) and a nuclear export signal (NES)-similar sequence, and is involved in the transport of target mRNAs [43]. In addition, it was reported that external stresses induce the translocation of hnRNP L to the cytoplasm for the regulation of mRNA stability and translation [31, 34, 35]. Taken together, these data indicate that hnRNP L may be transported to the cytoplasm under cell stress conditions, to regulate at the post-transcriptional level of those mRNAs which are important for cell survival. At the total protein level, not only hnRNP L but also p53 peak at 2 hours in NIH3T3 cells after drug treatment and remain steady. However, increased hnRNP L expression ceases, and hnRNP L protein levels seem to return to the basal levels, with long term exposure to high concentrations of etoposide (Figure 1A and 5F). We found that under these conditions, the total cell levels of hnRNP L proteins are the same at 0 and 18 hours points (Figure 5F), although we did not examine the cytoplasmic level of hnRNP L. Even though the cytoplasmic hnRNP L does not remain elevated under long exposure to etoposide, the increase in p53 remains constant. This may be due to post-translational modification of hnRNP L. It was suggested that phosphorylation of hnRNP L enhances or weakens the binding affinity for target mRNAs [44]. Thus, post-translational modification of hnRNP L under DNA damage conditions needs to be investigated. Another possibility is the contribution of some other factors to the control of p53 expression. Previously described post-translational and translation regulators of p53 must be considered, since a complex series of interactions may be involved in the delicate regulation of p53 expression.

We confirmed the effect of hnRNP L on cell cycle arrest after DNA damage. Cell cycle arrest was relieved at G2/M phase not at S phase when hnRNP L was silenced (Figure 5B). We can postulate several possibilities for this result. First, we assume that hnRNP L knock-down is more effective at G2/M phase when high induction of p53 protein occurs. We have already reported that p53 protein levels are higher in the G2/M arrested cell than in the G1 and S phase arrested cells, and the accumulation rate of p53 is enhanced at G2/M. In support of our assumption, IRES activity of human and mouse p53 mRNA is reported to be higher at G2/M [17, 38]. Another possibility is that hnRNP L knock-down influences the expression of other cell cycle regulators in addition to p53. As mentioned earlier, hnRNP L affects splicing, mRNA transport, translation, protein localization. Therefore, the potential roles of hnRNP L on other cell cycle regulators need to be considered.

We determined that hnRNP L has no impact on protein stability of p53 in both NIH3T3 and B16F10 cells (Figure 2C, 2D), but rather, stimulates the translation of p53 mRNA (Figure 2F). However, this finding is contrary to others that hnRNP L functions as an inhibitory binding partner of p53 protein in embryonic mouse stem cell (mESC) [45, 46]. We already know that the unique environment of each cell type affects cell specificity in transcription, splicing, and stability, and numerous genes have cell-type specific expression. Further, genes can function in a cell-type specific manner. For example, p18 is a known tumor suppressor, but accelerates cell growth of mESC in contrast to tumor and adult stem cells [47]. Therefore, factors that interact with hnRNP L may depend on cell type, which may in turn alter the functions mediated by hnRNP L. Furthermore, it is necessary to investigate and consider the complex and diverse functions of hnRNP L in diverse situations.

p53 is one of main regulatory genes for maintaining genomic integrity and cell survival. Abnormally low expression of p53 is associated with cell transformation and tumorigenesis, whereas its elevated expression induces cell death and inhibition of cell growth [48, 49]. Thus, it is important for p53 to be well regulated and expressed at the well-balanced level to maintain the integrity and survival of the cell. Because of this, regulatory mechanisms of p53 gene expression has been importantly studied. In case of p53 mRNA translation, several regulatory factors including miRNAs and RNA-binding proteins were found. miR-125b and miR-504 inhibit translation of p53 mRNA and apoptosis by binding to p53 3’UTR and increase tumorigenesis [14, 15]. It is also reported that hnRNP Q, RPL26, and PTB enhance p53 mRNA translation by binding preferentially to p53 5’UTR and elevate cell apoptosis and cell cycle arrest [12, 17, 29]. Likewise, hnRNP L functions as a positive regulator of p53 translation and increases cell apoptosis and cell cycle arrest under DNA damage condition. Under DNA damage condition or cancer developing environment, expression level, localization and activity of these factors seem to change and these changes regulate translation of p53 and cell fate. Though various cellular environments must be considered, it may be helpful for cancer therapy to control regulators of p53 translation.

## MATERIALS AND METHODS

### Plasmid and RNA interference

Bicistronic pRF p53 5’UTR vector for luciferase assay and pSk p53 5’UTR vector for *in vitro* binding assay were made by inserting the mouse p53 (accession no. NM_011640.3) 5’UTR as described previously [17]. Forward primer 5'-AAAAGCTTATGTCGCGGAGGCTGCTGC-3' and reverse primer 5'-CCGGATCCTTAGGAGGCGTGCTGAGC-3' (Macrogen, Seoul, Repulic of Korea) were used to make Flag-tagged hnRNP L.

The used siRNA duplex was as follows; Control 5’-CCUACGCCACCAAUUUCGUdTdT-3’ (Bioneer, Daejeon, Republic of Korea), Mouse hnRNP L #1 5’-GAUGAACUGUGAUCGAGUCdTdT-3’ (Bioneer). Mouse hnRNP L #2 5’-GAACGGAGUUCAGGCU AUGdTdT-3’ (Bioneer) is modified form from previously reported human hnRNP L siRNA [50].

### Cell culture and transient transfection

Mouse fibroblast NIH3T3 cells were cultured in Dulbecco's Modified Eagle's medium (DMEM; Hyclone) supplemented with 10% fetal bovine serum (FBS; Hyclone) and 1% penicillin/streptomycin. Cells were maintained in 5% CO_2_ at 37°C. The immortalized fibroblasts from p53/Mdm2 double-knockout mice, a gift from Dr. Jaewhan Song (Yonsei University, Seoul, Korea), and mouse melanoma B16F10 cells were grown in DMEM (Welgene) supplemented with 10% FBS (Welgene) and 1% penicillin/streptomycin. siRNAs and Flag vector were transfected into cells using the Neon microporation system (Invitrogen). At 24 h after this transfection, transfection of the pRF vectors was performed using Lipofectamine 2000 (Invitrogen) according to manufacturer's instructions. Cells were harvested after 24 h incubation.

### Protein expression and purification

Competent *E.coli* BL21(DE3) cells were transformed with vectors coding for Intein-hnRNP L and were grown. Proteins were induced by isopropyl β-D-1-thiogalactopyranoside (IPTG) at 18°C overnight. Cells were resuspended with Intein binding buffer (20mM Tris-HCl (pH8.0), 200mM NaCl, protease inhibitor) and lysed by sonicator. Cell extracts were incubated with chitin beads at 4°C overnight and then the beads were incubated with elution buffer (20mM Tris-HCl (pH8.0), 200mM NaCl, 50mM DTT) at 4°C overnight.

### Dual luciferase reporter assay

Cells harvested 24 h after transfection of the bicistronic pRF vector were lysed in 50 μl Reporter Lysis 5XBuffer (Promega). Luciferase activities of the samples were measured twice using the Dual-Luciferase Reporter Assay System (Promega) and luminometer according to the manufacturer's instructions.

### *In vitro* binding assay

pSK 5’UTR constructs including serially deleted p53 5’UTR (1–157, 87–157, 110–157, 87-109) and p53 5’UTR mutation were linearized with *Xba*I restriction enzyme. The linearized constructs were transcribed by T7 polymerase (Promega) in the presence of biotin-UTP (Roche) and then treated with DNase I (Promega) to remove DNA. The biotin conjugated RNAs were incubated with cell extracts for 30 mins at room temperature. Biotin-labeled RNAs and cell extracts were incubated with streptavidin beads (Thermo Scientific) at 4°C overnight. Proteins in the precipitates were detected by western blotting.

### Flow cytometry assay

NIH3T3 cells treated with 50 μM etoposide (Sigma) 24 h after transfection with control or hnRNP L siRNA were used for cell cycle analysis. Harvested cells were washed with and suspended in phosphate-buffered saline (PBS) containing 1% Bovine serum albumin (BSA). Cells were fixed and permeabilized in 95% ethanol containing 0.5% Tween-20 at 4°C overnight. After washing with PBS containing 1% BSA, cells were incubated with propidium iodide (PI) solution and RNaseA for 30 min at 37°C in the dark. DNA contents of cells were analyzed by flow cytometer (FACS caliber, Becton-Dickinson).

### Cell extract preparation and Immunoblotting

Cells were gently harvested and lysed with TNE buffer (50 mM Tris, 140 mM NaCl, 5 mM EDTA) containing Pierce^TM^ Protease Inhibitor (Thermo Scientific) and using sonication. Nuclear/cytosolic fractionations of NIH3T3 cells were conducted as previously described [17]. Proteins were separated on 12% SDS-PAGE gels and then transferred to nitrocellulose membrane. For protein detection, we used monoclonal anti-p53 (Cell Signaling), monoclonal anti-Flag (Sigma-Aldrich), monoclonal anti-hnRNP L (Abcam), polyclonal anti-GAPDH (Millipore), anti-Lamin B (Santa Cruz), anti-cleaved caspase3 (Cell Signaling), anti-phospho-Histone H2A.X (Ser 139) (Millipore) and Horseradish peroxidase (HRP) -conjugated mouse (Thermo Scientific) and rabbit (Promega) secondary antibodies. Enhanced chemiluminescence (ECL) was detected with the LAS-4000 system (FUJI FILM).

### Metabolic labeling

Metabolic labeling was performed as previously described [17]. In brief, NIH3T3 cells were seeded in 10-cm dishes. At 80% confluency, cells were washed with PBS and incubated for 1 h in methionine- and cysteine-free DMEM (Met-/Cys- DMEM) supplemented with 10% FBS and 1% antibiotics. The medium was then replaced with (Met-/Cys- DMEM) containing ^35^S-labeled methionine (^35^S-Met) and ^35^S-labeled cysteine (^35^S-Cys). After 1 h, the cells were harvested and lysed in buffer (20 mM Tris, 150 mM NaCl, 1 mM EDTA, 1 mM EGTA, 1% Triton). Immunoprecipitation was carried out with mouse IgG (Santa CruZ Biotechnology) or p53 mouse monoclonal antibody overnight.

### MTT assay

Cells were seeded onto 96 well plates at a density of 10,000 cells per well after transfection with control or hnRNP L siRNA, and then treated with 50 μM etoposide for 12 h or 18 h. 3-(4, 5-dimethylthiazolyl-2)-2,5-diphenyltetrazolium bromide (Tetrazolium MTT) solution was added to the cells and incubated for 2 h at 37°C. MTT crystals were solubilized in DMSO. Absorbance was measured at 570 nm (Infinite 200 NanoQuant, Tecan).

### Quantitative Real-time RT-PCR

To extract RNA from cells, we used TRI Reagent (Molecular Research Center, Cincinnati, OH, USA). Isolated RNA was reverse-transcribed using ImProm-IITM Reverse Transcription System (Promega). FastStart Universal SYBR Green Master (Rox) (Roche) was used for qRT-PCR with StepOnePlus Real-Time PCR System (Applied Biosystems, Carlsbad, CA, USA). The following primers were used: For the detection of mouse p53, forward 5’-GGATGCCCATGCTACAGAGGAGTCT-3’ and reverse 5’-GTCTGAGTCAGGCCCCACTTTCTTG-3’; mouse ribosomal protein L32 (mRPL32), forward 5’-AACCCAGAGGCATTGACAAC-3’ and reverse 5’-CACCTCCAGCTCCTTGACAT-3’; mouse β-actin, forward 5’-GGCACCACACCTTCTACAATG-3’ and reverse 5’-GGGGTGTTGAAGGTCTCAAAC-3’; p21, forward 5’-TTGCACTCTGGTGTCTGAGC-3’ and reverse 5’-TCTGCGCTTGGAGTGATAGA-3’; Mdm2, forward 5’-TGTGTGAGCTGAGGGAGATG-3’ and reverse 5’-ATCCTGATCCAGGCAATCAC-3’; Puma, forward 5’- GCTGAAGGACTCATGGTGAC-3’ and reverse 5’-CAAAGTGAAGGCGCACTG-3’; FLUC, forward 5’-CTCACTGAGACTACATCAGC-3' and reverse 5’-TCCAGATCCACAACCTTC GC-3'; mouse GAPDH, forward 5’-GCCATCAACGACCCCTTCATT-3’ and reverse 5’-GCTCCTGGAAGATGGTGATGG-3’;

### RNA-Immunoprecipitation

NIH3T3 cells were treated with or without 100 μM etoposide and lysed with RNAIP buffer (20mM Tris-HCl (pH7.5), 100mM KCl, 5mM MgCl_2_, 0.1% NP40, protease inhibitor). Mouse IgG or hnRNP L antibody was incubated with NIH3T3 cell lysates at 4°C overnight and then incubated with Protein G beads at 4°C for 4hr. We washed the beads 3 times with RNAIP buffer and isolated RNA using TRI reagent. RNA levels were quantified by qRT-PCR.

### TUNEL assay

siRNA transfected cells were grown on cover glass and exposed to 100 μM etoposide. Cells were fixed in 4% paraformaldehyde (Sigma-Aldrich) and permeabilized with 0.1% Triton X-100 solution (Sigma-Aldrich). Cells were rinsed with PBS, and apoptotic cells were detected by incorporation of fluorescein-12-dUTP at the fragmented DNA ends. TUNEL assay Kit (DeadEND Fluorometric TUNEL system, Promega) was used for labeling fragmented DNA according to the manufacturer's instructions. Nuclei of cells were stained with Hoechst 33342. The fluorescein-12-dUTP-labeled DNA was visualized by fluorescence microscopy (OLYMPUS 1×71).

### Statistical analyses

Data are shown as mean±SEM from independent experiments. Exact *n* value is represented in the figure legends. One-way, Two-way analysis of variance (ANOVA) and paired t-test were done using GraphPad Prism. *P*<0.05 was considered statistically significant. *P*<0.05, *P*<0.01, *P*<0.001, and *P*<0.0001 are indicated with *, **, ***, and ****, respectively.

## SUPPLEMENTARY MATERIALS FIGURES



## Abbreviations

hnRNP: heterogeneous nuclear ribonucleoprotein
IRES: Internal ribosome entry site
ITAFs: IRES trans-acting factors
RBP: RNA-binding protein
RLUC: *Renilla* luciferase
FLUC: *Firefly* luciferase
RNAIP: RNA-immunoprecipitation
TUNEL: Terminal deoxynucleotidyl transferase dUTP nick end labeling
NLS: Nuclear localization signal
NES: Nuclear export signal
mESC: Embryonic mouse stem cell
DMEM: Dulbecco's Modified Eagle's medium
FBS: Fetal bovine serum
PBS: Phosphate-buffered saline
PI: Propidium iodide
