# Supplementary material for: Heterogeneous nuclear ribonucleoprotein (hnRNP) L promotes DNA damage-induced cell apoptosis by enhancing the translation of p53
Source: Oncotarget. 2017 Apr 10;8(31):51108–22. doi: 10.18632/oncotarget.17003 (PMC5584235; doi:10.18632/oncotarget.17003)
Supplement: Supplementary file 1 [file oncotarget-08-51108-s001.pdf]

Heterogeneous nuclear ribonucleoprotein (hnRNP) L promotes DNA damage-induced cell apoptosis by enhancing the translation of p53

SUPPLEMENTARY FIGURES

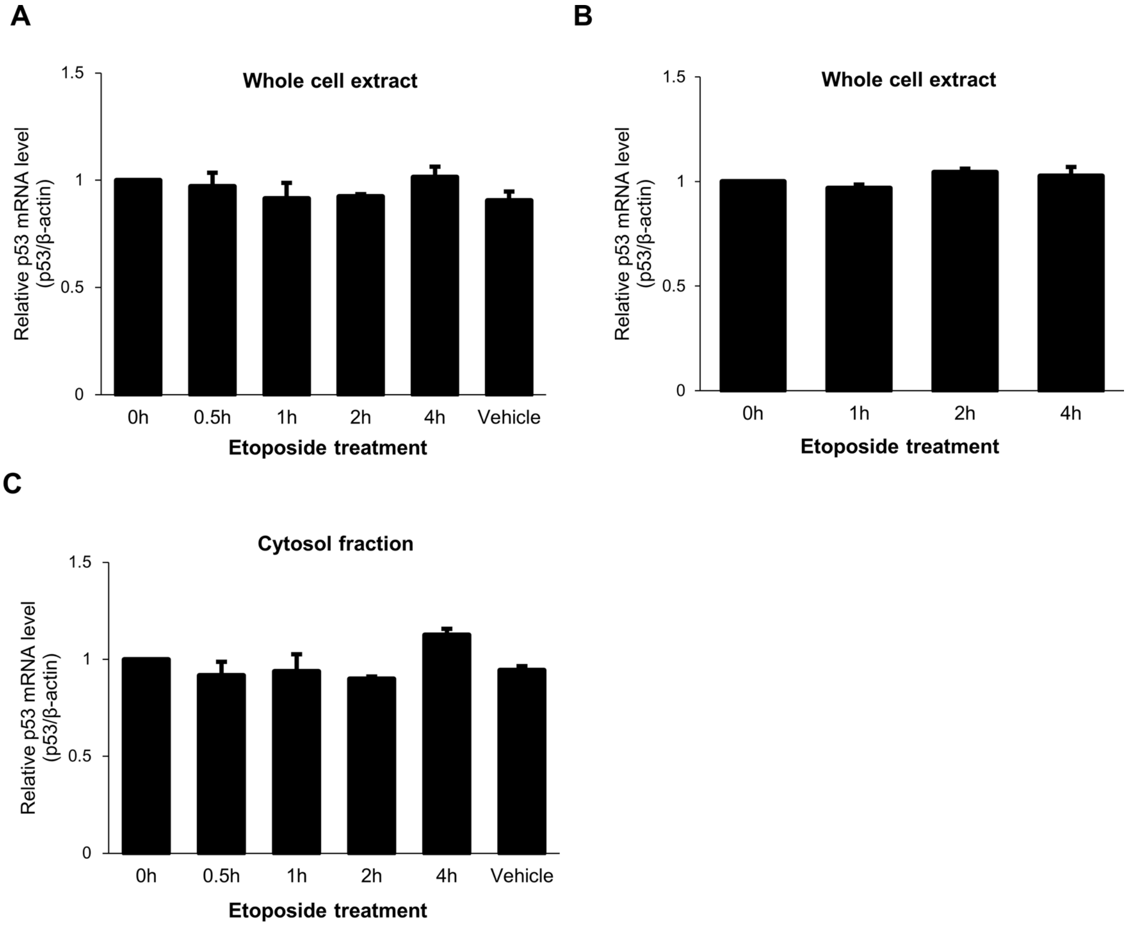

Supplementary Figure 1: p53 mRNA levels remain steady in presence of DNA damage-inducing drug. At the indicated time points after 100 µM etoposide treatment, the level of endogenous p53 mRNA in total NIH3T3 cell extracts **(A)**, B16F10 cell extracts **(B)** or NIH3T3 cytosolic lysates **(C)** was measured by qRT-PCR and normalized to β-actin mRNA. DMSO added for 4 h was used as vehicle control. p53 mRNA level at the 0 time point was set as 1. The bars represent the mean±SEM (n=4, 3, 2).

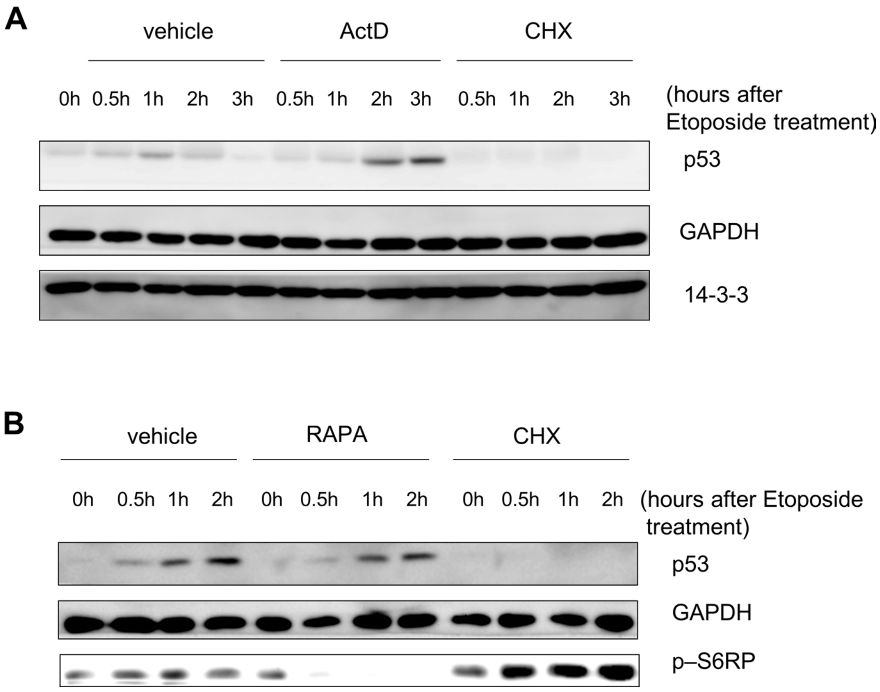

Supplementary Figure 2: Translation is necessary for induction of p53 under cell stress conditions. (A) Translation is essential to accumulate p53 proteins in DNA damage. p53 protein accumulation was determined by WB after treatment with 100 µM etoposide together with 5 mg/ml of actinomycin D (ActD) or 50 mg/ml of cycloheximide (CHX) for the indicated times. GAPDH and 14-3-3 were analyzed as loading controls. DMSO was used as a vehicle. **(B)** Cap-independent translation contributes to induction of p53 in presence of DNA-damaging drug. 100 µM etoposide was added to NIH3T3 cells with DMSO or rapamycin (RAPA) or cycloheximide (CHX).

A

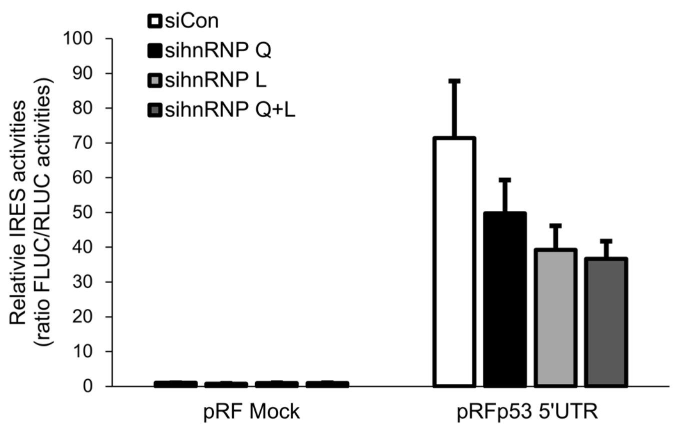

Supplementary Figure 3: IRES-mediated translation of p53 mRNA needs cooperation of hnRNP L and hnRNP Q. (A) Analysis of p53 5'UTR IRES activities. At 24 h after transfection with siRNAs, pRF mock or pRF mp53 5'UTR vector was transfected into NIH3T3 cells. IRES activity of pRF mock and control siRNA transfected cells was set as 1. The bars represent the mean±SEM (n=3).

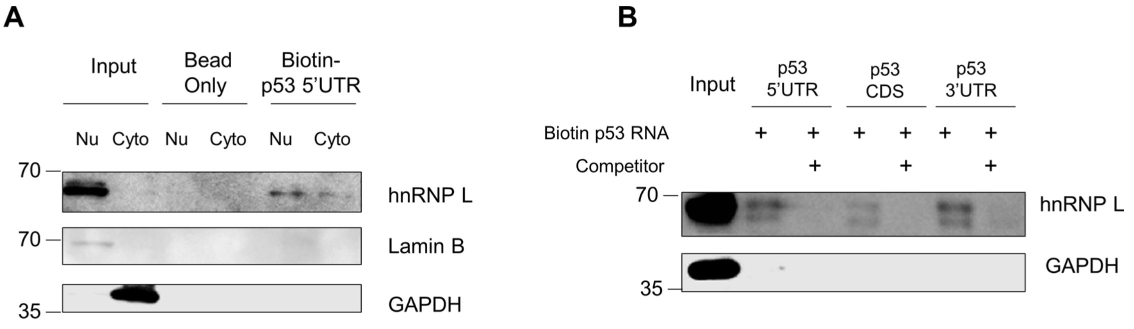

C

p53 5'UTR sequence
1 tttcccctcc cacgtgctca ccctggctaa agttctgtag
41 cttcagttca ttgggaccat cctggctgta ggtagcgact
81 acagttaggg ggcacctagc attcaggccc tcatcctcct
121 ccttcccagc agggtgtcac gcttctccga agactgg

Supplementary Figure 4: hnRNP L binds to p53 mRNA. (A) hnRNP L interacts with p53 5'UTR in both nucleus and cytosol. To verify whether hnRNP L binds to p53 5'UTR in cytosol, biotin-labeled p53 5'UTR was incubated with NIH3T3 cell nucleus or cytosol fraction. The interaction of p53 5'UTR and hnRNP L was verified by WB. GAPDH and Lamin B were used as cytosol marker and nucleus marker, respectively. **(B)** hnRNP L binds to untranslated region and coding region of p53. Bioin-labeled p53 RNAs were incubated with NIH3T3 extracts. GAPDH was used as negative control. **(C)** Mouse p53 mRNA 5'UTR sequence (NM_011640.3). Nucleotides from 87 to 109 are underlined in black and sequence written in red represents the putative binding region of hnRNP L.

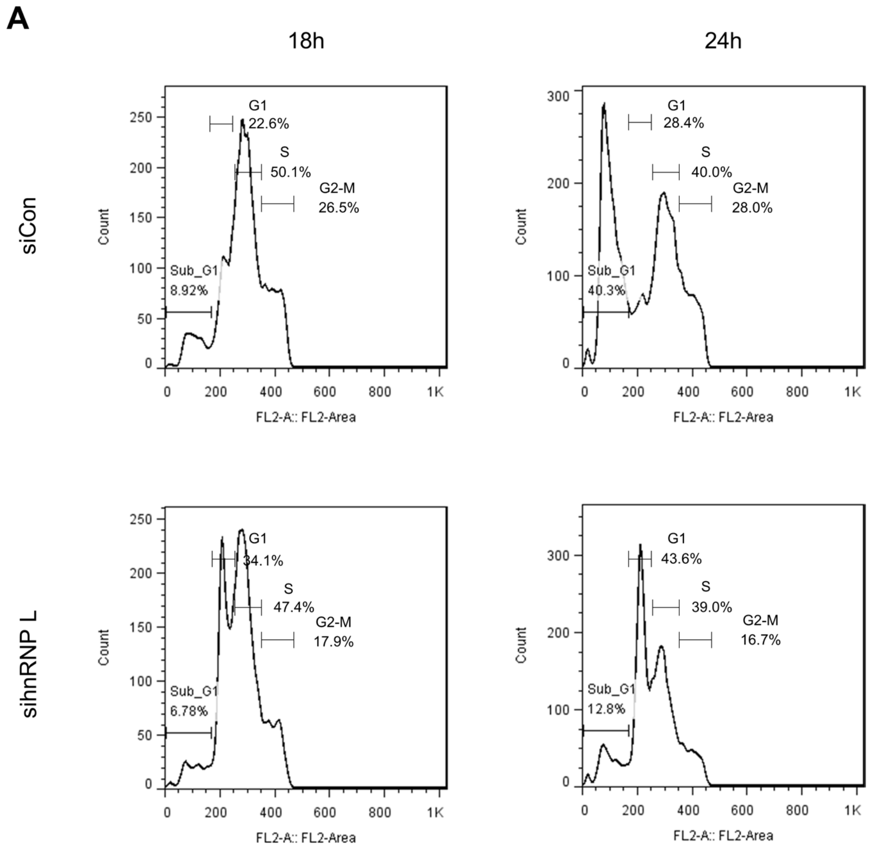

Supplementary Figure 5: Reduction of hnRNP L decrease cell cycle arrest in G2/M and cell death. (A) FACS analysis. At 24 h after transfection with control or hnRNP L siRNA, NIH3T3 cells were treated with 50 µM etoposide for the indicated times and stained with DNA dye, propidium iodide (PI). The data were analyzed by flow cytometry. Percentages of cells in sub G1, G1, S, and G2-M phases are shown. The sub G1, G1, S and G2-M fractions were quantified using FlowJo program. This is representative data from four independent experiments.
